# On the magnetic and crystal structures of NiO and MnO

**DOI:** 10.1107/S205252062400756X

**Published:** 2024-09-10

**Authors:** V. Pomjakushin

**Affiliations:** a Laboratory for Neutron Scattering and Imaging Paul Scherrer Institut (PSI) 5232Villigen PSI Switzerland; Oak Ridge National Laboratory, USA

**Keywords:** magnetic structures, Shubnikov groups, neutron diffraction, isotropy subgroups, multi-*k* structures

## Abstract

The magnetic and crystal structures of manganese and nickel monoxides have been studied using high-resolution neutron diffraction. The known 1*k*-structures based on the single propagation vector [½ ½ ½] for the parent paramagnetic space group *Fm*3*m* are forced to have monoclinic magnetic symmetry and are not possible in rhombohedral symmetry. However, the monoclinic distortions from the rhombohedral crystal metric allowed by symmetry are very small, and the explicit monoclinic splittings of the diffraction peaks have not been experimentally observed. We analyse the magnetic crystallographic models metrically compatible with our experimental data in full detail by using isotropy subgroup representation approach, including rhombohedral solutions based on the propagation vector star {[½ ½ ½], [−½ ½ ½], [½−½ ½], [½ ½ −½]}. Although the full star rhombohedral *R*_*I*_3*c* structure can equally well fit our diffraction data for NiO, we conclude that the best solution for the crystal and magnetic structures for NiO and MnO is the 1*k* monoclinic model with the magnetic space group *C*_*c*_2/*c* (Belov–Neronova–Smirnova No. 15.90, UNI symbol *C*2/*c*.1′_*c*_[*C*2/*m*]).

## Abstract 2

The magnetic and crystal structures of manganese and nickel monoxides have been analysed for possible multi-*k* solutions and compared with high-resolution neutron diffraction data.

## 1. Introduction

Since the first neutron diffraction studies of manganese and nickel monoxides (Shull *et al.*, 1951 ▸), interest in the magnetic and crystal structures of MnO and NiO has been maintained [see *e.g.* experimental studies (Cheetham & Hope, 1983 ▸; Lee *et al.*, 2016 ▸) and theoretical first principal calculations (Schrön *et al.*, 2012 ▸; Lim *et al.*, 2016 ▸) and references cited therein]. It is well established experimentally that the cubic paramagnetic crystal structure 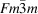 transforms metrically to rhombo­hedral 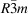 at the transition to an antiferromagnetically ordered structure below the Néel temperature *T*_N_. The antiferromagnetic structure is well described with one propagation vector **k**_1_ = [½  ½  ½] (in cubic crystal metric) and the moment directed perpendicular to **k**_1_. This type of structure corresponds to the monoclinic magnetic space group (MSG) *C*_*c*_2/*m* [No. 12.63 Belov–Neronova–Smirnova (BNS)], whose properties and relation to the parent cubic group are shown in Table 1 ▸. The second high-symmetry monoclinic MSG is *C*_*c*_2/*c* (No. 15.90) and it allows spin components both parallel and perpendicular to **k**_1_ (the *x*-component in *C*_*c*_2/*c* settings is perpendicular to cubic **k**_1_, and the *z*-component makes an angle ∼35° to the **k**_1_ direction). Experimentally, one does see rhombohedral distortion in the title compounds, but the monoclinic distortions from the 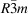 crystal metric allowed by symmetry are very small and the explicit monoclinic splittings of the diffraction peaks have not been experimentally observed. Thus, a quite logical idea would be try to find the solutions in rhombohedral symmetry that would fit the experimental data. The only rhombohedral MSG permitting non-zero magnetic moments on Ni/Mn for a one-**k**_1_ structure is 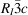 (1*k*) with a single Wyckoff position for Mn, but this structure cannot fit experimental diffraction intensities at all because it gives zero intensity for the most intense magnetic peak (½ ½ ½). However, the rhombohedral structures based on several propagation vectors (arms) from the propagation vector star {**k**_1_} = {[½ ½ ½], [−½ ½ ½], [½ −½ ½], [½ ½ −½]} could, in principle, be compatible with experimental data. In this paper we study possible rhombohedral and monoclinic solutions for multi-*k* magnetic structures based on several arms from the star {**k**_1_} and compare the models with high-resolution neutron diffraction data.

## 2. Experimental

Neutron powder diffraction measurements were carried out on the high-resolution powder diffractometer for thermal neutrons (HRPT) (Fischer *et al.*, 2000 ▸) at the SINQ neutron spallation source (Paul Scherrer Institute, Switzerland), using high-resolution mode (δ*d*/*d* > 10^−3^) with neutron wavelength λ = 1.155 Å and high-intensity mode with neutron wavelength λ = 1.886 Å. The refinements of the structure parameters were carried out using the *FullProf* (Rodríguez-Carvajal, 1993 ▸) program, with the use of its internal tables for neutron scattering lengths. The symmetry analysis was performed using the *ISODISTORT* tool based on the *ISOTROPY* (Stokes & Hatch, 1988 ▸; Campbell *et al.*, 2006 ▸) software, the software tools of Bilbao Crystallographic Server (Aroyo *et al.*, 2011 ▸) and the *BasiRep* program (Rodríguez-Carvajal, 1993 ▸). The magnetic structure figures were prepared using the *VESTA* (Momma & Izumi, 2011 ▸) software program.

## 3. Symmetry analysis

**Figure 1:**
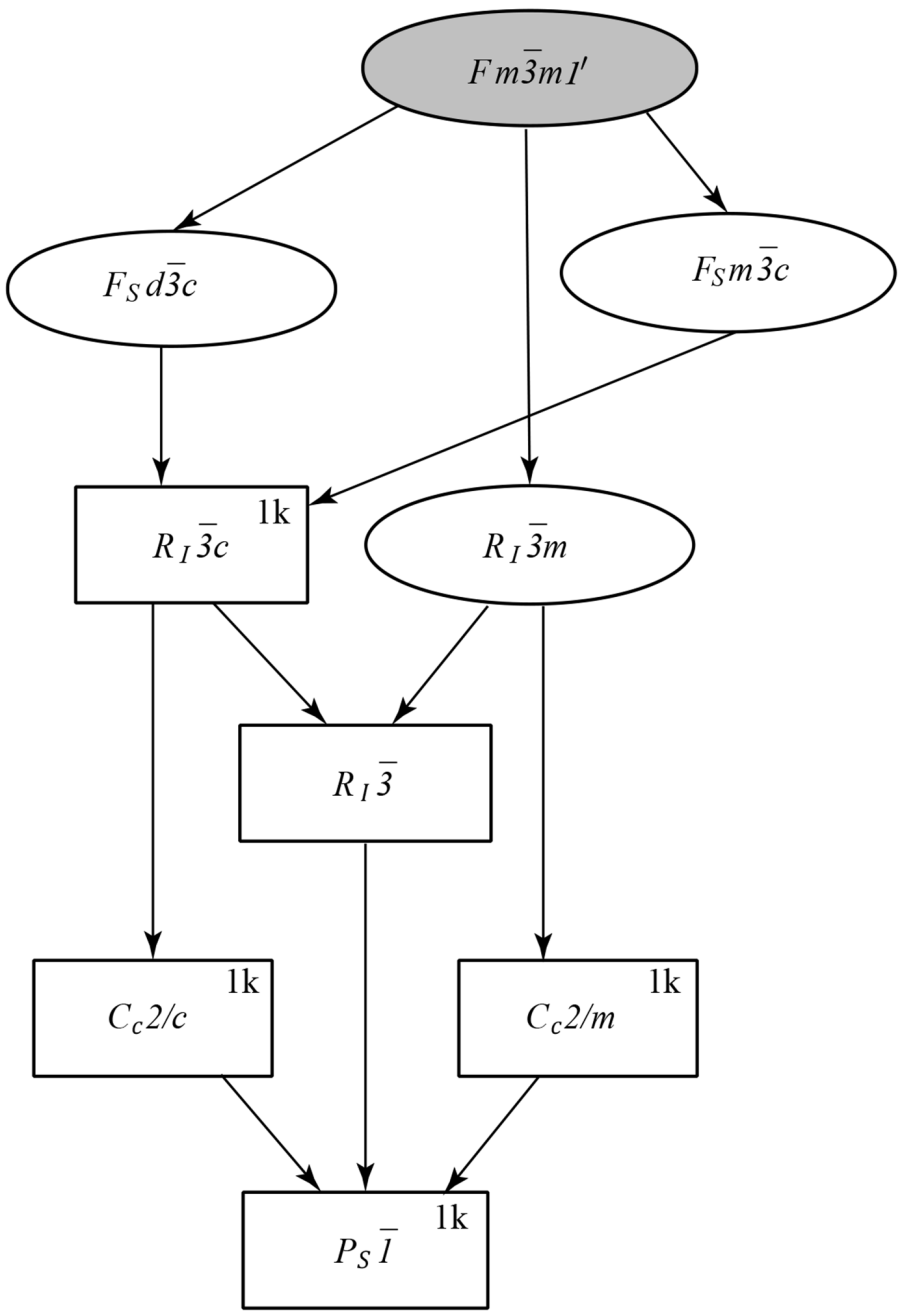
Group–subgroup graph for 4*k* structures based on the propagation vector star generated by [½ ½ ½] in space group 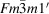 with the magnetic atom in the 4*a* (0,0,0) position. The possible 1*k* structures are indicated by label 1*k*. Only cubic, rhombohedral and monoclinic groups are shown, whose crystal metrics are compatible with the experiment.

The paramagnetic crystal structure of MnO and NiO is 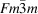 with Mn or Ni in the 4*a* (0,0,0) position and oxygen in the 4*b* (½, ½, ½) position. All magnetic peaks are indexed with the propagation vector **k**_1_ = [½ ½ ½], which is the L-point of the Brillouin zone. The decomposition of the magnetic representation for the 4*a* position into irreducible representations (irreps) reads 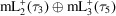 with the dimensions of irreps being 1D and 2D, respectively. The nomenclature for the irreps is given in accordance with Campbell *et al.* (2006 ▸) and Aroyo *et al.* (2011 ▸), with Kovalev (1993 ▸) notation in the parentheses. For the single propagation vector 1*k* structure, there are four subgroups shown in Fig. 1 ▸. The one-dimensional irrep 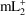 results in the magnetic structure 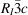 with the moments parallel to **k**_1_. The subgroups generated by the irrep 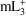 as the primary order parameter result in the magnetic structure with the moments perpendicular to **k**_1_ in MSG *C*_*c*_2/*m*, whereas two other groups *C*_*c*_2/*c* and 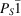 allow the presence of irrep 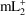 as a secondary order parameter, which allows a spin component along the propagation vector.

We note that for the 1*k*-case, *C*_*c*_2/*m* and 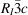 are maximal magnetic subgroups of the parent grey Shubnikov group 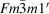. The basis transformation from cubic to monoclinic cells for both *C*_*c*_2/*m* and *C*_*c*_2/*c* reads: (½, ½, −1), (½, −½, 0), (−1, −1, 0). In MSG *C*_*c*_2/*c*, Ni and O are in the 4*c* (000 | m_*x*_,0,m_*z*_) and 4*b*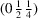 positions. In MSG *C*_*c*_2/*m*, Ni and O are in the 4*a* (000 | 0,m_*y*_,0) and 4*d* (0 ½ ¼) positions, respectively. The (*ab*) monoclinic plane is perpendicular to the propagation vector **k**_1_, so in *C*_*c*_2/*m* the allowed spin direction is only perpendicular to **k**_1_. The propagation vector **k**_1_ and the *a* and *c* axes lie in the same plane with an ideal pseudocubic monoclinic angle (between *a* and *c*) β = arccos[−1/(3)^1/2^] ≃ 125.264°, and an angle between **k**_1_ and the *c* axis 270 − β ≃ 144.736°.

For the multi-*k* structures of the propagation vector star {[½ ½ ½], [−½ ½ ½], [½−½ ½], [½ ½ −½]} of 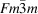, the number of possible subgroups is significantly larger. In the primitive basis, the arms of the star are {[½ ½ ½], [0 0 ½], [0 ½ 0], [½ 0 0]}. As we will show below from the diffraction experiments, the crystal metric in the AFM state is rhombohedral. For that reason we exclude possible tetragonal and orthorhombic MSGs and their subgroups because the crystal metric from experiment cannot be fitted to the orthorhombic one. The eight MSGs that are left to consider are shown as a tree graph in Fig. 1 ▸. Four subgroups labelled with 1*k* are MSGs allowed for a single propagation vector **k**_1_. We note that although the labels for the groups for 1*k*, 2*k*, 3*k* and 4*k* cases are the same, the magnetic structures are different. Three rhombohedral MSGs are 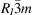, (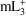, P22), 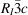 (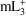, P21), and 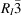 (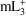, C56), with the active primary irrep and direction of the order parameter shown in the parenthesis in accordance with Campbell *et al. *(2006 ▸). The basis transformation is (−1, 0, −1), (0, 1, 1), (2, 2, −2), and the single 4*a* site in 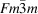 is split into 18*e* and 6*a* positions with spin components 18*e* (m_*x*_,0,0), 6*a* (0,0,0); 18*e* (m_*x*_,2m_*x*_,m_*z*_), 6*a* (0,0,m_*z*_) and 18*e* (m_*x*_,m_*y*_,m_*z*_), 6*a* (0,0,m_*z*_) for the above-listed rhombohedral groups, respectively. The last two MSGs allow the presence of the secondary irrep 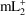. The first MSG does not allow the non-zero magnetic moment on all magnetic atom sites and hence can be excluded. Interestingly, the MSG 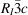 generated by the primary irrep 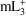 with three active **k** vectors can be also generated by the irrep 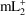, C7 with four **k** vectors. The magnetic structures in both cases are the same. The MSG 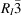 is a direct subgroup of 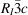 with identity basis transformation and has the same positions for Mn/Ni and O atoms, but due to the lost of the mirror plane, the point group allows a general direction of the magnetic moment in the 18*e* position.

The full star monoclinic subgroup *C*_*c*_2/*c* (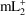, S2) is constructed in a similar way to the 1*kC*_*c*_2/*c*, but the single 4*c* position is split into three special positions 4*c* (m_*x*_,0,m_*z*_), 4*d* (m_*x*_,0,m_*z*_) and 8*f* (m_*x*_,m_*y*_,m_*z*_). If the spin components are constrained to be the same for all three positions, then the magnetic 4*k* structure is equivalent to 1*k* structure.

## 4. Neutron diffraction results

### 4.1. Crystal structure

**Figure 2:**
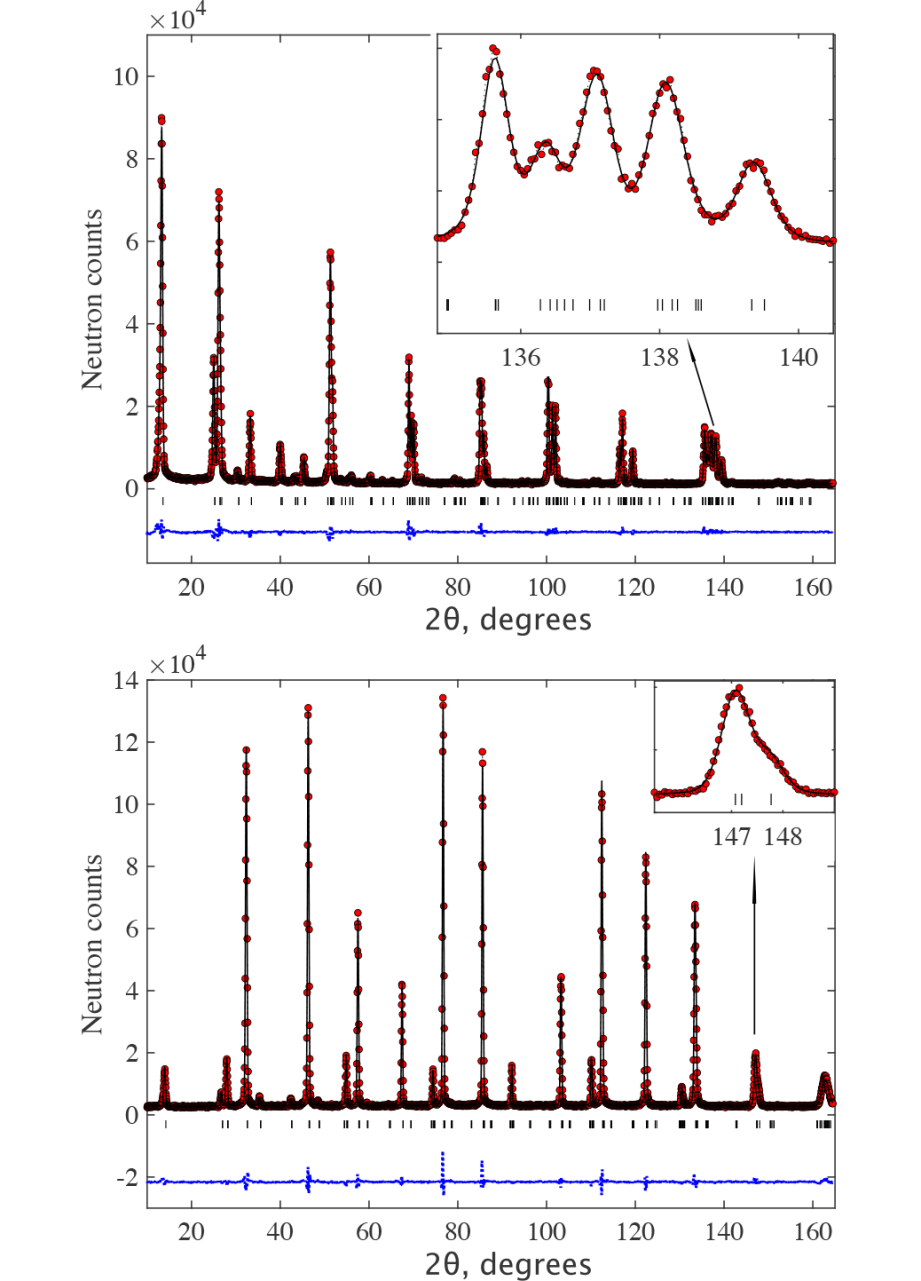
The Rietveld refinement pattern and the difference plot of the neutron diffraction pattern for MnO (top) and NiO (bottom) at *T* = 2 K measured on the HRPT with the wavelength λ = 1.1545 Å. The line is the refinement pattern based on the magnetic model *C*_*c*_2/*c* shown in Table 1 ▸. The row of ticks show the Bragg peak positions. The insets show fragments of diffraction pattern at high angles.

The diffraction patterns were collected at a base temperature of *T* = 2 K for all samples and at some selected temperatures below and above the Néel temperature *T*_N_ = 115 K for MnO. The diffraction pattern for NiO taken at room temperature (∼300 K), is similar to the one at base temperature, because *T*_N_ = 525 K is significantly higher than room temperature. Fig. 2 ▸ shows the diffraction patterns of MnO and NiO at 2 K together with the refinement curves. One can see that the rhombohedral distortion in MnO is very large: the group of five peaks around 2θ = 140° is mainly the splitting of the single cubic peak (711)/(551). In space group 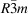, they are (321), (045), (137), (309) and (2,0,11). For NiO the rhombohedral splitting is about ten times smaller than for MnO, but is clearly seen as an 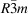 doublet (404), (0,0,12) from the splitting of the (444) cubic peak, around 2θ = 147°. The rhombohedral distortions from the parent cubic metrics can be defined as the ratio δ_R_ = [(6)^1/2^ − *c*/*a*]/(6)^1/2^ and amount to δ_R_ ≃ 1.9 × 10^−3^ and 16 × 10^−3^ for NiO and MnO, respectively, where *a* and *c* are rhombohedral lattice constants.

The structure can be further refined in *C*_*c*_2/*c*, leading to additional monoclinic splitting of the above peaks. The fit to the monoclinic group is converged with the metrics shown in Table 1 ▸. We note that all the atoms preserve their special positions in the *C*_*c*_2/*c* symmetry, so there are no extra structure parameters to refine, allowing a convergent fit. The monoclinic distortion can be refined as well. Despite the fact that the structures can be refined in *C*_*c*_2/*c*, there is no apparent peak splitting and the improvement of the goodness-of-fit χ^2^ is marginal. The refined monoclinic distortion from rhombo­hedral metric corresponds to the splitting of the Bragg peaks (shown in the inset of Fig. 2 ▸) δ*d*/*d* ≃ 3 × 10^−4^ and 6 × 10^−4^ for the NiO doublet around 2θ = 147° and MnO around 2θ = 139.2°, respectively. The metric transformation from 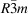 to *C*_*c*_2/*c* reads: (−1, −2, 0), (−1, 0, 0), (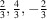). This transformation results in the following monoclinic metric that preserves rhombohedral symmetry: *A* = (3)^1/2^*a*, *B* = *b*, 
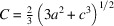
, β = 180 − 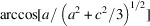, where capital letters stand for the monoclinic metric. This ‘ideal’ value of β, derived by the above formula, is also listed in Table 1 ▸. We think that the refined monoclinic deviations from the rhombohedral symmetry may suffer from systematic errors related to profile parameters. From the viewpoint of high-resolution neutron diffraction, the crystal metric is indistinguishable from the rhombohedral one.

### 4.2. One-**k**_1_ magnetic structure

All magnetic Bragg peaks can be indexed with a single propagation vector **k**_1_ in 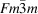 settings. More precisely, due to very strong rhombohedral distortions, the propagation vector that fits the experimental peak positions is 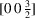 in 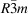 settings with the basis transformation from the cubic group shown in Table 1 ▸. The one-*k* rhombohedral magnetic subgroup 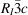 has the magnetic ion in the 6*b* Wyckoff position, which allows a spin component only parallel to 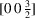, and gives the structure with ferromagnetic (*ab*) layers with antiparallel orientation of neighbouring layers along *c*. This type of structure does not fit the experiment because it does not explain the first magnetic peak (½ ½ ½) (in cubic setting) or (0 0 3) in the above rhombohedral group.

**Figure 3:**
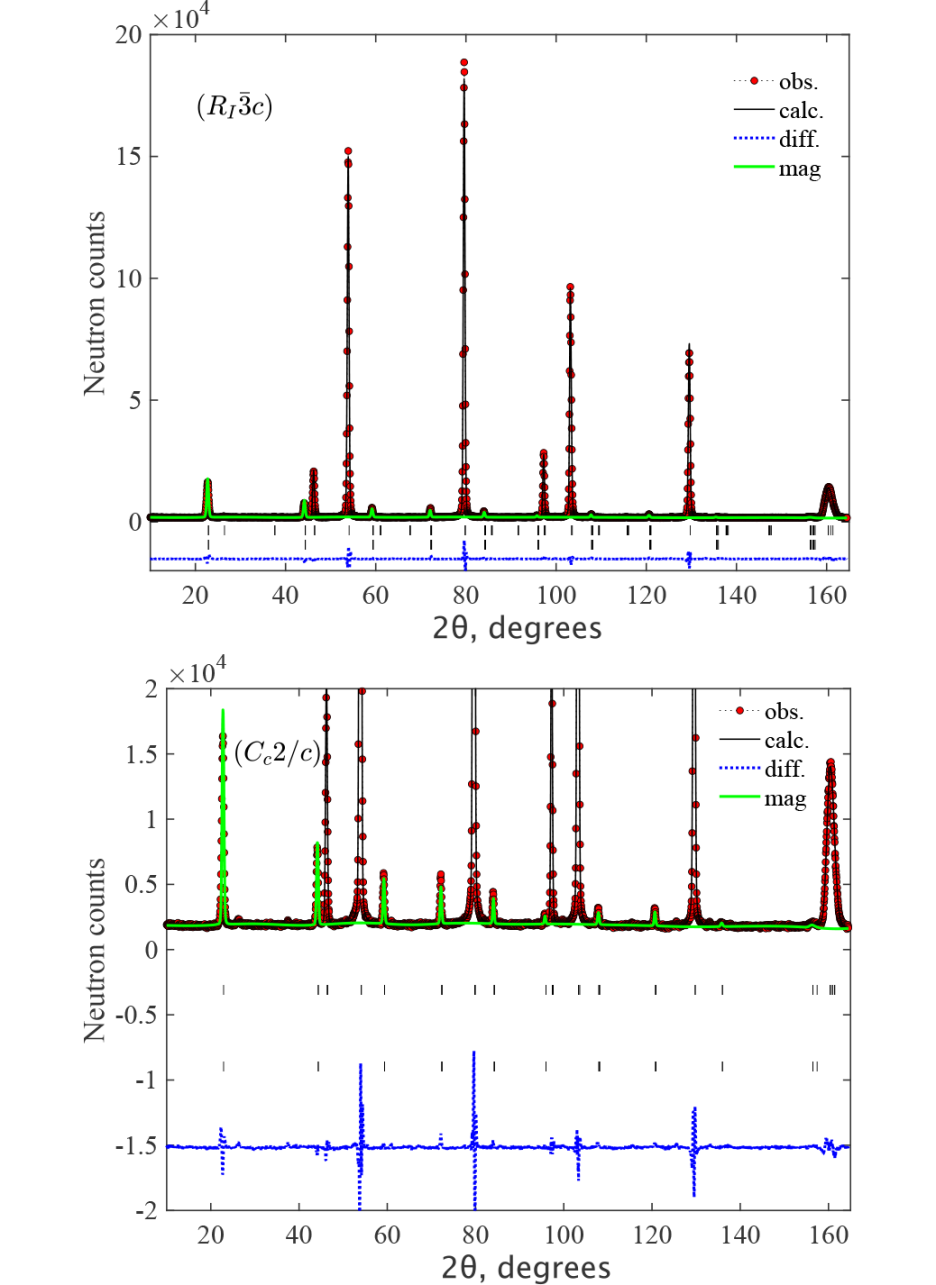
The Rietveld refinement pattern and the difference plot of the neutron diffraction pattern for NiO at *T* = 2 K measured at HRPT with the wavelength λ = 1.886 Å. The lines are the refinement patterns based on the magnetic model 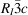 (top) and *C*_*c*_2/*c* (bottom) shown in Table 1 ▸. The magnetic contributions are shown by the green lines. The rows of ticks show the Bragg peak positions for crystal (upper) and magnetic (lower) structures. The difference between the observed and calculated intensities is shown by the dotted blue line. Since both refinements are visually identical, we zoom the bottom *C*_*c*_2/*c* figure to better show the magnetic contribution. The minor unindexed peaks are due to λ/2, as discussed in the text and illustrated in Fig. 8 ▸.

**Figure 5:**
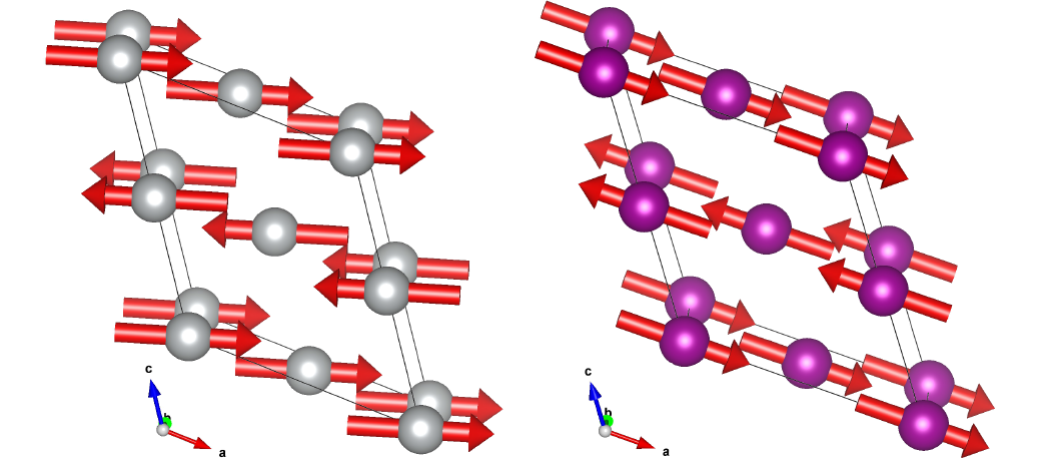
Magnetic structure of NiO (left) and MnO (right) in MSG *C*_*c*_2/*c*. The details of the magnetic models are given in Table 1 ▸.

The data are very well fitted in the *C*_*c*_2/*c* MSG (Fig. 3 ▸) with the structure parameters shown in Table 1 ▸. The directions of moments along specific monoclinic axes are dictated only by the symmetry of the magnetic space group and are conditional because the crystal metric does not show any explicit deviations from the rhombohedral one, according to our experimental data. There was no convergence of the fit with the m_*z*_ component released for MnO, so it was fixed to m_*z*_ = 0. Since the experimental data do not show monoclinic distortions, the absence of a spin component along the **k**_1_ vector raises the symmetry of MSG to *C*_*c*_/2*m*, where the allowed spin direction is only m_*y*_, which is perpendicular to **k**_1_, similar to m_*x*_. The fit in MSG *C*_*c*_/2*m* gives the same goodness-of-fit and the same magnetic moment for MnO. The description of the magnetic structure for both samples is given in *C*_*c*_/2*c* in Table 1 ▸. The formal description of the magnetic structures in the mcif format is given in supporting information. Figs. 3 ▸ and 4 ▸ show the neutron diffraction patterns together with the fits to the magnetic structure models. For MnO, the figure shows the diffraction patterns below and above the Néel temperature *T*_N_ = 115 K, where one can observe the progressive development of rhombohedral distortions as the temperature decreases to 100 K and 2 K. This effect is particularly noticeable at diffraction angles around 2θ ≃ 90° and 140°, evidenced by an increased splitting of the single cubic peaks that were observed above the Néel temperature at 125 K. The presence of the short-range magnetic correlations just above *T*_N_ is also clearly seen as a broad (½ ½ ½) peak at 2θ ≃ 20°. The magnetic structures are illustrated in Fig. 5 ▸. The magnetic moment sizes are close to the expected spin-only values 2 μ_B_ and 5 μ_B_ for NiO and MnO, respectively.

The structure description in 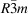 given in Table 1 ▸ does not correspond to any rhombohedral MSG but is equivalent to the magnetic structure with MSG *C*_*c*_2/*c*, with the crystal metric fixed as explained in Section 4.1. In this description, the crystal structure is refined in space group 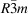, whereas the magnetic structure is defined in *P*1 with rhombohedral centring translations (*R*1). In the rhombohedral metric, the moment components are along and perpendicular to the *c* axis. For NiO, there is a spin component parallel to the propagation vector, whereas it is absent for MnO. This type of representation is useful for comparison with the literature data and the multi-*k* model considered in Section 4.3.

### 4.3. Multi-*k* magnetic structures in NiO

Two rhombohedral groups 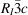 [No. 167.108 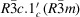 (UNI symbol)] and its subgroup 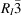 (No. 148.20 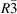) allow a spin component on both magnetic sites 18*e* and 6*a*. We performed a simulated annealing (SA) search (Kirkpatrick *et al.*, 1983 ▸; Rodríguez-Carvajal, 1993 ▸) of the full diffraction profile for possible solutions in most general rhombohedral MSG 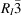 for both NiO and MnO samples. The SA search starts from random values of the free parameters, and we have repeated the search more than several hundred times, so we are confident that we have not lost any solutions.

**Figure 6:**
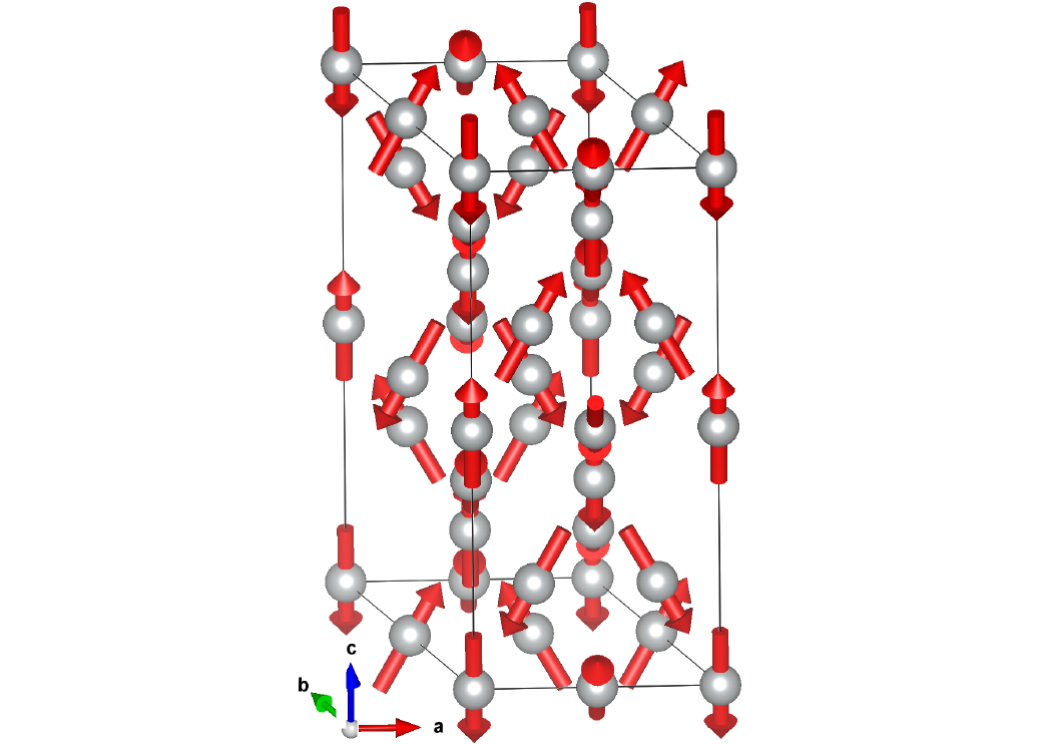
Magnetic structure of NiO multi-*k* MSG 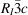. Details of the magnetic model are given in Table 1 ▸.

For the MnO, there is no reasonable multi-*k* rhombohedral solution in 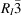 that would be compatible with the 1*k* structure based on the goodness-of-fit. For NiO, we have found two structures that give a goodness-of-fit similar to the one-*k* structure in the *C*_*c*_2/*c* 1*k* model. The results of the SA search are shown in Table 1 ▸. One of the structures has ‘good’ sizes of the magnetic moments, about 2 μB, which is shown on the first line for the 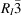 model SA search in Table 1 ▸. One can see that the components along *x* and *y* are related by a factor of two, suggesting the 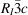 symmetry. Using these initial values of the parameters, we have performed a standard least squares (LSQ) fit, but the fit did not converge for all parameters released. We attempted a restricted fit under the assumption of the same moment sizes on both Ni sites using spherical coordinates with a fixed ratio of m_*x*_/m_*y*_ = 0.5, but there was no convergence for the spherical θ angle to the *z* axis. So, in the final LSQ fit, the angle was fixed to −35° (close to the value from the SA search). The error bars correspond to the θ variation between −30° and −40°. The formal description of the structure is given in supporting information. The illustration of the fit quality can be seen in Fig. 3 ▸. The magnetic structure in this multi-*k* model in MSG 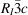 is shown in Fig. 6 ▸. The magnetic structure description with respect to the atomic positions and the crystal metrics is the same in both MSG 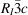 and its subgroup 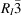, provided that we correlate m_*y*_ = 2m_*x*_ in the latter. In supporting information, we give the description of the structure in 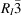 and, for completeness, we also provide an mcif file with the equivalent description of this structure in MSG 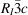.

## 5. Discussion

The magnetic structure in MnO can be described using two different one-*k* MSGs, *C*_*c*_2/*c* and *C*_*c*_2/*m*, which give different magnetic moment directions with respect to the same monoclinic metrics in these groups. Since our diffraction data do not show significant deviations from the rhombohedral crystal metrics, we cannot unambiguously assign the MSG symmetry. We note that the above MSGs are not group–subgroup related. Therefore, we use the less restricted monoclinic MSG *C*_*c*_2/*c* for the magnetic structure description for both samples. Furthermore, as we have shown, our data for both MnO and NiO samples are not sensitive to the orientation of the magnetic moment in the plane perpendicular to the rhombohedral *c* axis in 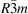 SG (direction of **k**_1_ = [½ ½ ½] in parent SG 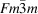) – we can only determine the components of the magnetic moment along and perpendicular to **k**_1_.

**Figure 7:**
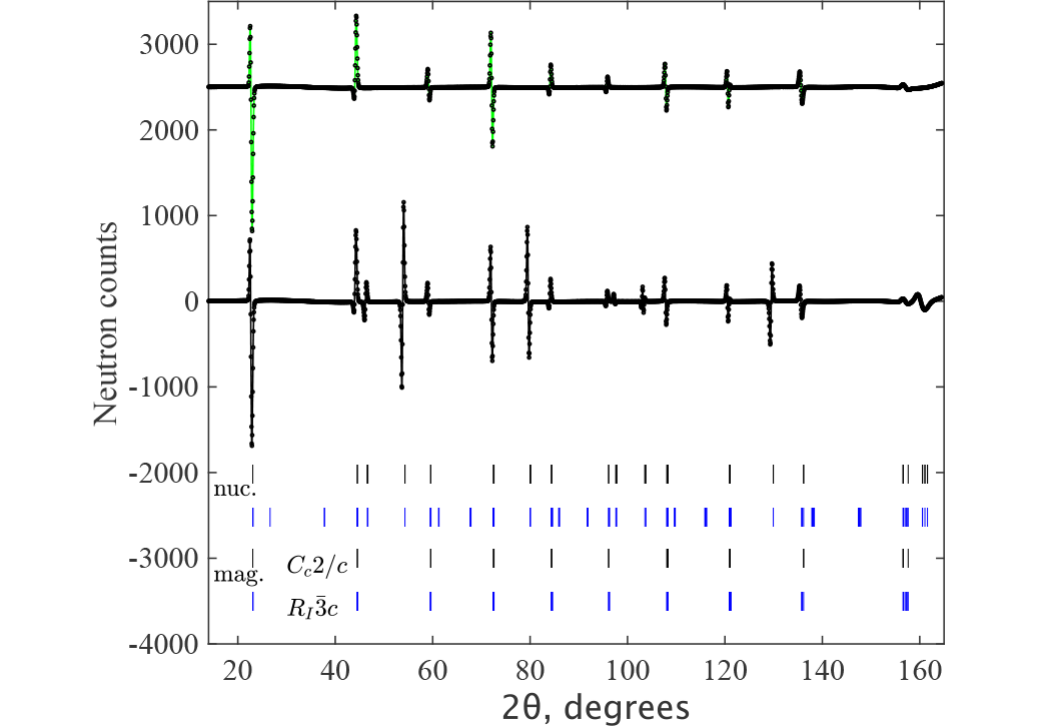
The difference between the calculated neutron diffraction patterns shown in Fig. 3 ▸ for the models 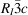 and *C*_*c*_2/*c* with refinement parameters shown in Table 1 ▸ for NiO at *T* = 2 K. The line around zero level is the overall difference between the calculated patterns. The line shifted up to the count level 2500 is the difference of pure magnetic contributions between the two models. The rows of ticks show the Bragg peak positions for crystal (upper) and magnetic (lower) structures.

The neutron diffraction patterns allow two very different magnetic structure models for NiO, which fit the data equally well. The directions of the magnetic moments in the monoclinic one-*k* structure *C*_*c*_2/*c* and the rhombohedral 4*k* structure 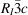 are practically orthogonal to each other. In the one-*k* structure, the Ni moments form ferromagnetic layers with the moment direction approximately perpendicular to the propagation vector **k**_1_ and stacked antiferromagnetically (AFM) along **k**_1_. In the multi-*k* magnetic structure, the moments of the neighbouring Ni atoms are approximately AFM coupled. These two structures are completely incompatible with each other and we think that the 4*k* structure 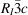 is a false solution and is only possible because of the very small rhombohedral distortions in NiO, as well as the small moment value. We cannot formally disregard this magnetic structure because it gives the same goodness-of-fit, but we like to show the reasons for excluding this model. Fig. 7 ▸ shows a comparison between the models. The lines show the difference between calculated diffraction profiles for the models 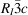 and *C*_*c*_2/*c*. The allowed magnetic Bragg reflections are localized at approximately the same 2θ values, but due to rhombohedral distortion, all peaks are split in the larger unit cell in the 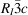 model. For instance, the first magnetic peak at 22.5°, which is a single (001) peak in *C*_*c*_2/*c*, becomes a doublet in 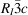 (101)/(003) with (101) at slightly lower 2θ. Thus, the (003) peak has zero intensity in 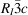, but all calculated intensity belongs to the (101) peak. Conversely, the single (001) peak in *C*_*c*_2/*c*, metrically corresponding to (003), describes all the experimental intensity. This repartitioning of the peak intensities results in the up-down shape of the calculated intensity difference clearly seen at all magnetic peak positions in Fig. 7 ▸. Although the models in Figs. 5 ▸ and 6 ▸ may be indistinguishable by neutron diffraction, they almost certainly have very different total energies. *Ab initio* calculations can and should be used to select the correct model in cases like NiO.

**Figure 8:**
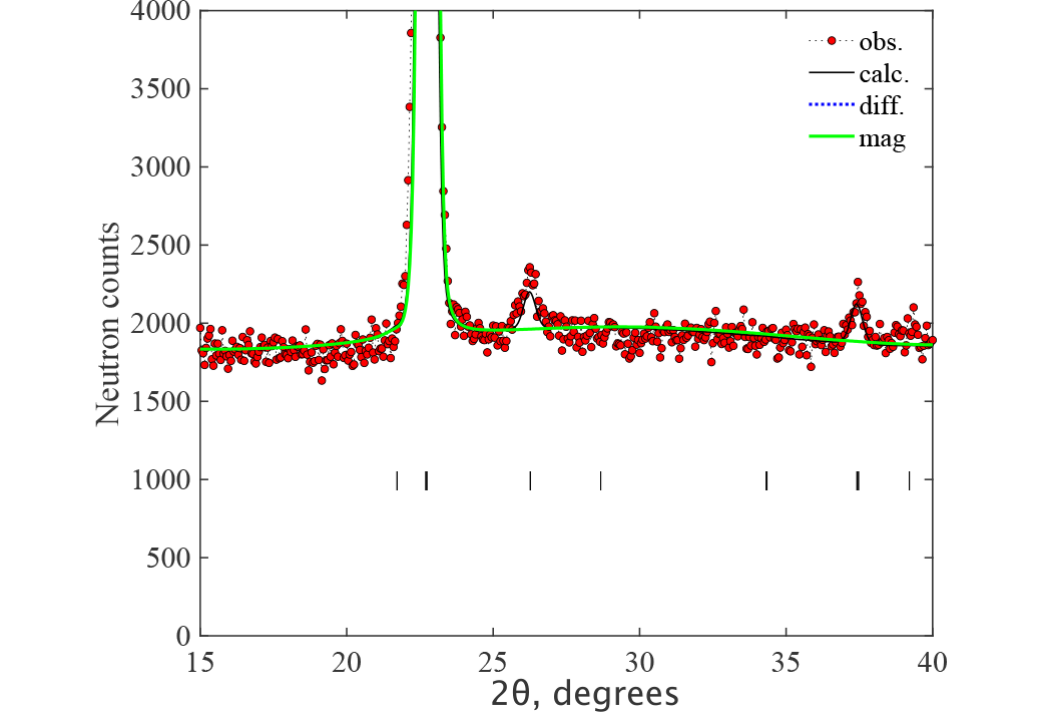
A portion of the Rietveld refinement pattern for NiO at *T* = 2 K measured on the HRPT with wavelength λ = 1.886 Å, demonstrating the presence of a very small but visible contribution (about 0.05%) of λ/2 at 2θ about 26° and 37° corresponding the strongest Bragg peaks (111) and (311) in cubic metric. The black line is the refinement pattern *C*_*c*_2/*c* (bottom) shown in Table 1 ▸. The magnetic contribution is shown by the green line. The row of ticks shows the Bragg peak positions.

The presence of the extra Bragg peaks in larger unit cell of 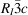 could potentially confirm the multi-*k* magnetic structure, although these peaks have only nuclear contributions in 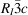. Close inspection of the diffraction pattern shows that a few peaks are indeed present (Fig. 8 ▸), but they originate from a very small admixture of the λ/2 component in the neutron beam and their intensities are ideally refined in the one-*kC*_*c*_2/*c* model. Ironically, we did not realize the presence of 0.05% of λ/2 in the neutron beam, which theoretically should not be present for the (511) reflection of the Ge monochromator used for λ = 1.886 Å. Thus, the presence of these extra peaks was initially attributed to the multi-*k* structure, and we attempted to find the solutions in all possible multi-*k* structures shown in the subgroup tree (Fig. 1 ▸).

In MnO, due to significantly larger rhombohedral distortions, one can clearly see that the intensity distribution over the doublets like (101)/(003) definitively allows us to reject the rhombohedral model. We believe that the exchange inter­actions in both compounds are similar, and also on these grounds, the monoclinic model is the only acceptable solution for the magnetic structure in NiO. An interesting previous study (Goodwin *et al.*, 2006 ▸) reports that the monoclinic, collinear structure of MnO is favored when considering the total (Bragg + diffuse) neutron scattering, and it also resolves the direction of the moment in the (111) planes. The solution proposed is similar to the one we have found, but with a different monoclinic symmetry, *C*2. This group cannot be a magnetic subgroup of the parent grey MSG, but it can, of course, be modelled using the lowest MSG 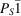.

We would like to note that distinguishing between multi-*k* and one-*k* structures from diffraction experiments is difficult, if not impossible, unless there is a magnetostructural distortion associated with the different symmetries of the multi-*kversus* one-*k* cases. The point is that different *k* vectors from the propagation vector star do not interfere with each other, and their partial contributions are simply added to the Bragg peaks located at the same *Q*-value in powder diffraction. The one-*k* structure contributes to the Bragg peaks located at the same *Q*, as in the multi-*k* case, and we think that in most cases one can find one-*k* and multi-*k* models that provide the same integral intensity at the same *Q*. However, this is not always possible if we keep the same magnetic space group symmetry. For instance, in our case, the 1*k* and 4*k* magnetic structures with very different arrangements of the magnetic moments in the same MSG 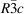 (mL^2+^) result in different magnetic Bragg peak intensities, and the 1*k* structure can be definitely rejected. However, it is true that one can find the one-*k* structure in monoclinic MSG *C*c**2/*c*, which gives the same powder diffraction intensities in the absence of crystal metric distortions. The situation is not much easier in single-crystal diffraction where, instead of powder averaging, we always have magnetic domains for the one-*k* structure that mimic the multi-*k* case.

In some cases, one can distinguish between the models using single-crystal neutron diffraction in an applied magnetic field because multi-*k* and one-*k* structures will be modified differently [see, for instance, Puphal *et al.* (2020 ▸)]. Alternatively, one could potentially affect the population of the magnetic domains by applying uniaxial pressure, thus identifying the one-*k* model. Additionally, there is an example of inelastic neutron scattering on powder samples where the authors claim that they can distinguish multi-*q* from single-*q* states (Paddison *et al.*, 2024 ▸) from the analysis of the magnetic excitations in linear spin-wave theory.

## 6. Summary

We have performed experiments using high-resolution powder neutron diffraction on MnO and NiO monoxides with Néel temperatures (*T*_N_) of 115 K and 525 K, respectively, in order to refine their crystal and magnetic structures. The structures below *T*_N_ are generated by a propagation vector **k**_1_ = [½ ½ ½] in the parent paramagnetic cubic SG 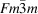. The rhombohedral distortions from the paramagnetic parent cubic metrics are clearly seen in our diffraction data and were refined in SG 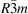, amounting to 1.9 × 10^−3^ and 16 × 10^−3^ for NiO and MnO, respectively. In accordance with the rhombohedral symmetry, there are eight possible magnetic space subgroups for the one-*k* and multi-*k* structures. Among them, there are the three most symmetric MSGs: 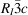, *C*_*c*_2/*m* and *C*_*c*_2/*c*. The refined monoclinic distortions from the rhombohedral metric are very small, about a few 10^−4^, and can be disregarded, implying that the orientation of the magnetic moment in the plane perpendicular to the rhombohedral *c* axis cannot be inferred from our data.

We have found that the best antiferromagnetic structure, which fits both MnO and NiO, is the 1*k* structure with MSG *C*_*c*_2/*c* (BNS No. 15.90, UNI symbol *C*2/*c*.1′_*c*_ [*C*2/*m*]). In this structure, the moments form ferromagnetic layers with the moment direction approximately perpendicular to the propagation vector **k**_1_ and stacked antiferromagnetically along **k**_1_ with magnetic moment sizes *m* = 1.94 μ_B_ and 4.61 μ_B_ for NiO and MnO, respectively. The same magnetic configuration can be described equally well in MSG *C*_*c*_2/*m* for MnO. For NiO, the multi-*k* rhombohedral structure in MSG 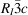, which is very different from the *C*_*c*_2/*c* structure, can equally well fit our diffraction data with similar value of *m*. However, we conclude that this is a false solution and is only possible because of the very small rhombohedral distortions and the small magnetic moment value in NiO.

In conclusion, we would like to note that this is not an uncommon case where the crystal distortions due to magnetoelastic coupling below the magnetic transition are small or even undetectable in diffraction experiments. Since a paradigm such as ‘one propagation vector at the magnetic transition is enough’ is still strong in the neutron scattering community, some high-symmetry multi-*k* magnetic structures are even not considered as possible structure solutions, which could, however, fit the experimental data equally well.

## Supplementary Material

mcif NiO_P7_C_c2c. DOI: 10.1107/S205252062400756X/gar5002sup1.txt

Four magnetic test mCIFs: . DOI: 10.1107/S205252062400756X/gar5002sup2.zip

## Figures and Tables

**Figure 4:**
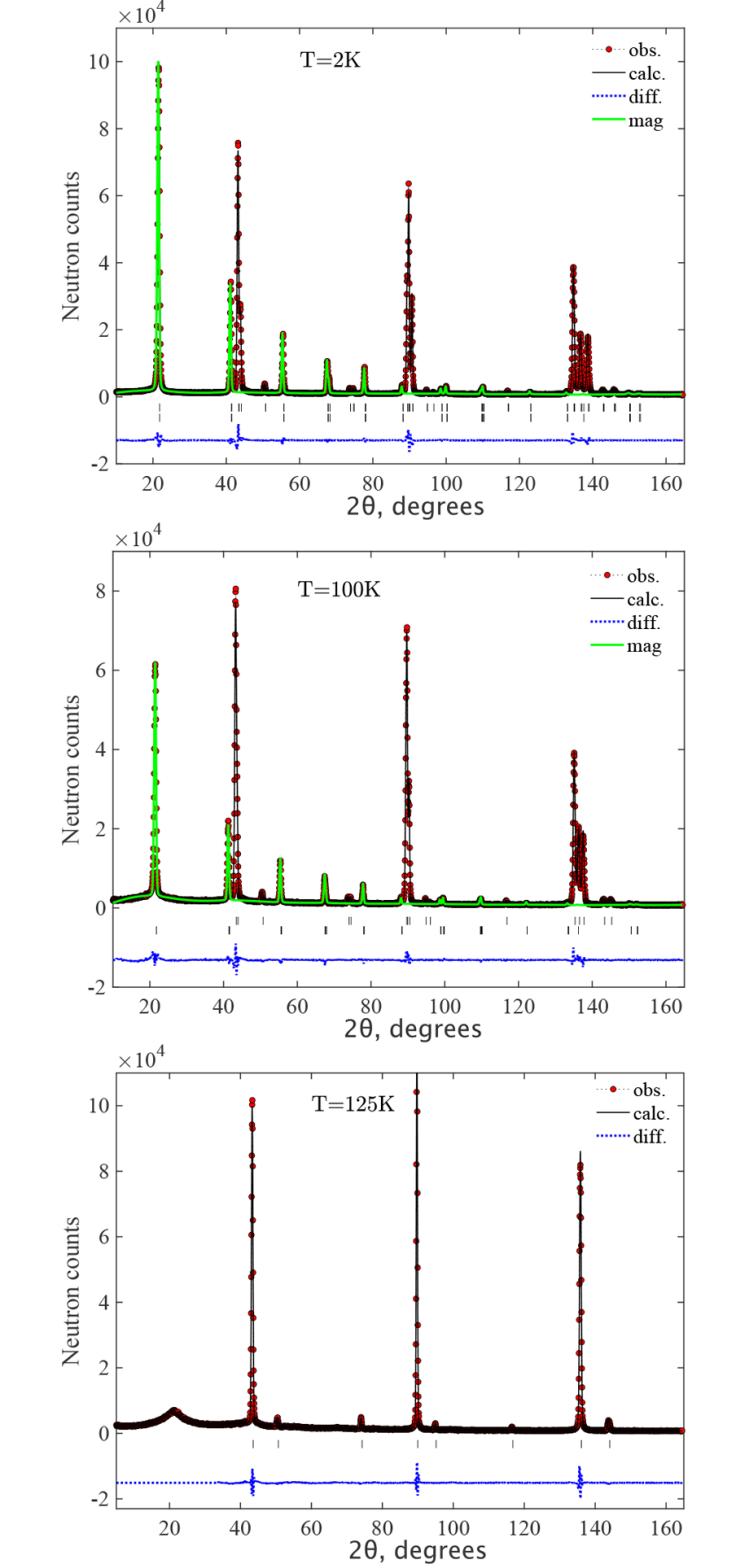
The Rietveld refinement pattern and the difference plot of the neutron diffraction pattern for MnO at *T* = 2 K (top), 100 K (middle) and 125 K (bottom) measured at HRPT with the wavelength λ = 1.886 Å. The lines are the refinement patterns for MSG *C*_*c*_2/*c* below the Néel temperature for *T* = 100 K and paramagnetic SG 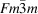 for 125 K. The magnetic contributions are shown by the green lines. The rows of ticks show the Bragg peak positions for crystal (upper) and magnetic (lower) structures. The difference between the observed and calculated intensities is shown by the dotted blue line.

**Table:** The conventional reliability *R* factors (%) (Rodríguez-Carvajal, 1993 ▸) are also given after SG symbol for the fits with λ = 1.155 Å. The reliability factors on the separate lines, labelled *R*_p_, *R*_wp_, *R*_exp_ and χ^2^ are for the fits with λ = 1.886 Å, aimed at detailing the magnetic structure, with the magnetic moment components provided in the previous line. The fits in rhombohedral space group 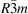 with the propagation vector 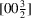 for the magnetic structures give an equivalent description of the structures. The metric transformation from cubic to monoclinic cells reads: (½, ½, −1), (½, −½, 0), (−1, −1, 0) and to 1*k* rhombohedral reads: (−½, ½, 0), (0, −½, ½), (1, 1, 1). The monoclinic unit-cell parameters derived from the rhombohedral metric are shown after the comma. Ni and O occupy 3*a* (000) and 3*b* (00½) Wyckoff positions in space group 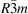, respectively. The magnitude of the magnetic moment is denoted by *m*, with m_*x*_, m_*y*_ and m_*z*_ representing its components along their respective axes of their MSGs. The magnetic moment has both components perpendicular and parallel to the propagation vector. In the magnetic space group *C*_*c*_2/*c*, Ni and O are in 4*c* (000 | m_*x*_,0,m_*z*_) and 4*b* (0 ½ ¼) positions. The moment directions in monoclinic groups along *x*, *y*, and *z* axes correspond to (½, ½, −1), (1, −1, 0) and (−1, −1, 0) in cubic metric, respectively. The Wyckoff positions in the multi-*k*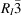 MSG are 18*e* (½, 0, 0) and 6*a* (0, 0, 0) for Ni1 and Ni2 and 18*d* (½, 0, ¼) and 6*b* (0, 0, ¼) for O1 and O2, respectively. Here *m* is the size of the magnetic moment; θ and ϕ are spherical angles with *c* and *a* axes, respectively in MSGs 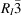.

	NiO	MnO
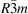 χ^2^	8.94, 9.59, 5.84, 2.70	8.71, 9.87, 7.18, 1.89
*a* (Å)	2.95006 (3)	3.14980 (7)
*c* (Å)	7.21235 (9)	7.59158 (16)
m_*x*_, m_*z*_, *m* (μ_B_)	1.840 (9), 0.63 (3), 1.943 (9)	4.75 (1), 0, 4.75 (1)
*C*_*c*_2/*c* χ^2^	8.84, 9.01, 5.87, 2.36	8.46, 9.54, 7.14, 1.79
*a* (Å)	5.10263 (8), 5.10967	5.45583 (15), 5.45657
*b* (Å)	2.95045 (6), 2.95007	3.15006 (9), 3.15035
*c* (Å)	5.89158 (11), 5.89263	6.22984 (15), 6.233451
β (°)	125.1722 (9), 125.3159	125.6659 (13), 125.7027
m_*x*_, m_*z*_, *m* (μ_B_)	2.28 (2), 0.77 (4), 1.946 (9)	4.61 (1), 0, 4.61 (1)
*R*_p_, *R*_wp_, *R*_exp_, χ^2^	3.67, 5.10, 2.31, 4.86	4.51, 6.04, 3.18, 3.61

**Table:** 

	SA search for NiO in MSG 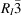	
Magnetic moments	Ni1 (18*e*)	Ni2 (6*a*)
m_*x*_, m_*y*_, m_*z*_, *m* (μ_B_)	0.5582, 1.1093 −1.6018, 1.8678	0, 0, 2.0484, 2.0484
	−0.5386, −1.1026, 0.2224, 0.9805	0, 0, −3.4317, 3.4317
*R* factors	3.65, 5.27, 2.31, 5.19	

	LSQ fit in MSG 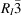	
*a*, *c* (Å)	5.90046 (6), 14.42656 (18)	
*m* (μ_B_), ϕ, θ (°)	1.976 (9), 90, 35 (5)	1.977 (9), 0, 180
m_*x*_, m_*y*_, m_*z*_ (μ_B_)	−0.65 (8), −1.31 (17), 1.6 (1)	−1.977 (9) 0 0
*R*_p_, *R*_wp_, *R*_exp_, χ^2^	3.72, 5.37, 2.31, 5.38	
